# Kalman Filters in Geotechnical Monitoring of Ground Subsidence Using Data from MEMS Sensors

**DOI:** 10.3390/s16071109

**Published:** 2016-07-19

**Authors:** Cheng Li, Rafig Azzam, Tomás M. Fernández-Steeger

**Affiliations:** 1 Chengdu Engineering Corporation Limited, Chengdu 610072, China; 2 Department of Engineering Geology and Hydrogeology, RWTH Aachen University, Aachen 52064, Germany; azzam@lih.rwth-aachen.de; 3 Department of Applied Geosciences, TU Berlin University, Berlin 10587, Germany; fernandez-steeger@tu-berlin.de

**Keywords:** Kalman filter, ground subsidence, rotation matrices, accelerometer, inclinometer

## Abstract

The fast development of wireless sensor networks and MEMS make it possible to set up today real-time wireless geotechnical monitoring. To handle interferences and noises from the output data, Kalman filter can be selected as a method to achieve a more realistic estimate of the observations. In this paper, a one-day wireless measurement using accelerometers and inclinometers was deployed on top of a tunnel section under construction in order to monitor ground subsidence. The normal vectors of the sensors were firstly obtained with the help of rotation matrices, and then be projected to the plane of longitudinal section, by which the dip angles over time would be obtained via a trigonometric function. Finally, a centralized Kalman filter was applied to estimate the tilt angles of the sensor nodes based on the data from the embedded accelerometer and the inclinometer. Comparing the results from two sensor nodes deployed away and on the track respectively, the passing of the tunnel boring machine can be identified from unusual performances. Using this method, the ground settlement due to excavation can be measured and a real-time monitoring of ground subsidence can be realized.

## 1. Introduction

Due to the fast development of Micro Electro Mechanical Systems (MEMS) and the optimization of sensor cost, size and energy consumption in the last two decades, Wireless Sensor Networks (WSNs) has been progressively entered many areas such as disaster monitoring [1,2,3] and industrial sensing [4,5]. A WSN consists of spatially distributed sensor nodes to monitor various conditions such as temperature, pressure, humidity, sound, vibration, pollutants and motion. In this sense they allow us to enter Internet of Things (IoT) and provide physical information about our environment to users where ever needed. Nowadays, real-time remote surveillance of environmental conditions has been achieved and a much more comprehensive understanding of the variations of natural environments over time can be obtained.

**Figure 1:**
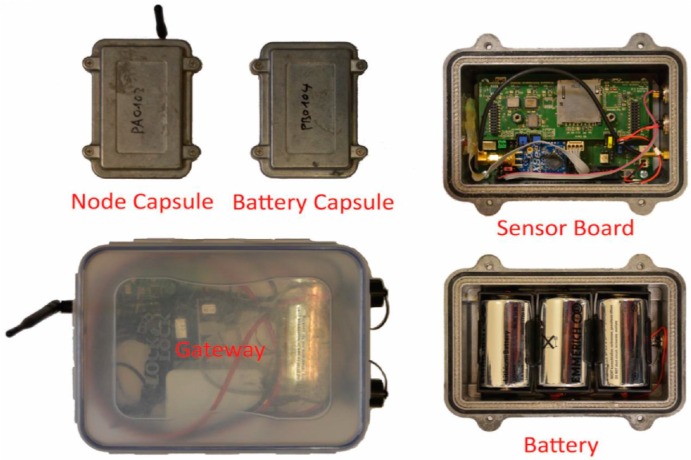
Components of X-SLEWS (From [11]). The node capsule comprises a base board for basic operation and an add-on board for sensing and storing function. The battery capsule allocated with three 3.6-V batteries provides the power supply to the node capsule while measuring and data transmitting. The gateway receives and saves the retrieved data from all the nodes in the network and broadcasts the updated clock periodically to connected nodes.

From 2007 to 2011, the Department of Engineering Geology and Hydrogeology at RWTH Aachen University and its partners carried out a joint project namely SLEWS (a Sensor based Landslide Early Warning System), aiming at developing a prototype of an alarm and early warning system for landslides. SLEWS was funded by GEOTECHNOLOGIEN, which is a geoscientific research and development program supported by the German Federal Ministry for Education and Research (BMBF) and the German Research Foundation (DFG). A self-organizing and multi-hop wireless monitoring network was created thereby [6,7]. This wireless monitoring system and its predecessor system X-SLEWS [8] have been applied to monitor several kinds of natural hazards and geotechnical activities such as tilting of rock towers deformation [9], ground subsidence due to ground improvement measures [10], tunneling construction [11], and landslide monitoring [12]. Figure 1 exhibits the main components of the X-SLEWS network. The node capsule comprises a base board for processing and radio and an add-on board for sensing and backup data storage. The add-on board is easily replaced by another one with different sensors to allow fast adoption to distinct tasks. The battery capsule allocated with three 3.6-V batteries provides the power supply to the sensor node capsule, separating node and energy source allows also to exchange energy sources or add modules for energy harvesting like solar panels. The gateway receives and stores the retrieved data from all the nodes in the network and broadcasts the updated clock periodically to connected nodes. It bears also a raspberry PI with a server to control communication and data storage and can be additional equipped with a data up-link via 3G modem for remote operation and monitoring [8,11].

The X-SLEWS wireless monitoring system was applied in a one-day field test on top of the construction site of the South Hongmei Road super highway tunnel in Shanghai, and positional information of the sensor nodes has been obtained based on the measurements from embedded MEMS accelerometers and inclinometers. Both of the derived normal vectors based on the output of the MEMS sensors contain interferences and noises and thus a data filter was needed for a good estimate. In the following we will briefly introduce the sensors and measuring principle applied. Then the processing of sensor data using rotation matrices will be described. Finally, the usage of the Kalman filter for data estimation is described in detail and the results will be discussed.

## 2. Applied MEMS Sensors

Each sensor node has been equipped with a 3-axis accelerometer and two orthogonal 1-axis inclinometers, both of which are produced by Murata Electronics Oy (previous called VTI Technologies, Vantaa, Finland) and are mounted on an add-on board. In Table 1 a generic data sheet for the two sensor is shown.

**Figure 2:**
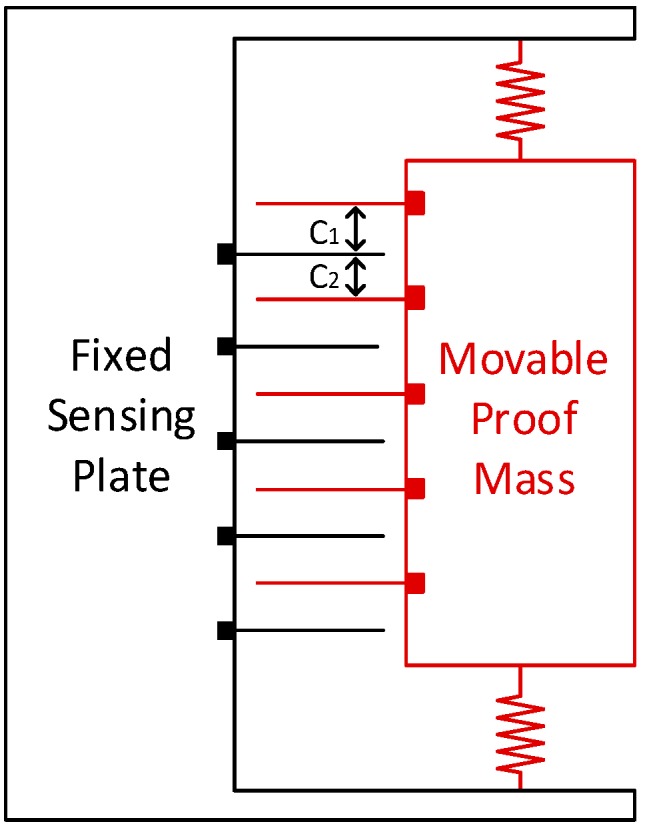
Sketch of a MEMS Accelerometer (Modified from [14]). The sensing plate is fixed onto the sensor board and the movable proof mass is suspended by the restoring spring. The proof mass will drift downward when a linear acceleration points upwards or the Earth’s gravitational field points downwards.

Basically, MEMS Accelerometers and inclinometers share the same principle to observe acceleration. A sketch of a MEMS accelerometer is shown below in Figure 2. The movable proof mass is suspended by the restoring springs, while the sensing plates are fixed onto the sensor board. The accelerometer is sensitive to the linear acceleration of the sensor and the earth gravitational field. When the sensing plate deflects from the original position, the change in capacitance (C1 and C2) between the fingers of the proof mass and sensing plates can be converted into a voltage signal, which is subsequently digitized to digital value, from which the acceleration along the axis can be expressed through a given formula. Due to different precision of the sensors, the output of the accelerometer and inclinometer is substituted into respective formulas to obtain the acceleration value [13].

**Figure 3:**
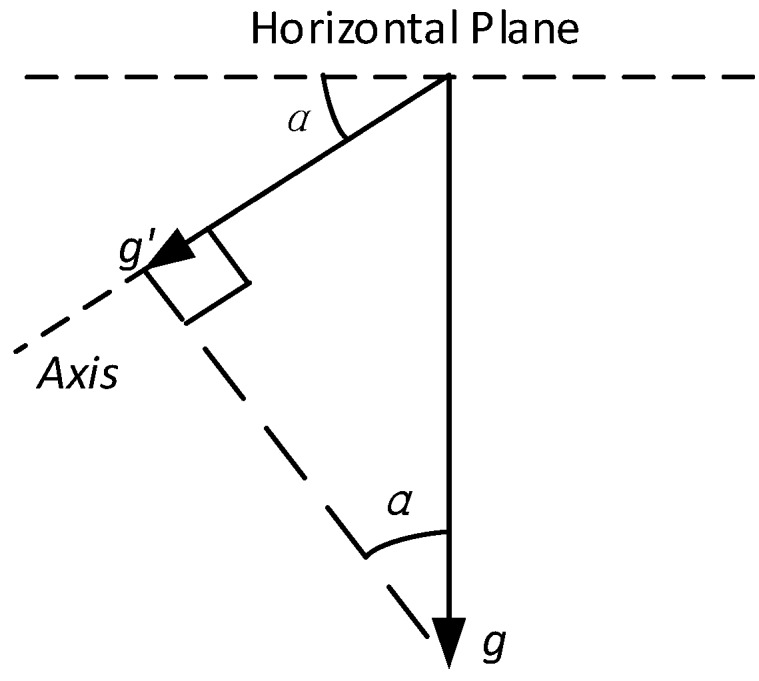
Measurement of Inclination from the Accelerometer. The accelerometer measures the value of $g^{\prime }$, which represents for the weight of the earth’s gravitational acceleration g in the axial direction on the sensor board. The tilt angle of the axis ∝ with respect to the horizontal plane can be calculated via an inverse trigonometric function: $\propto =\text{arcsin}\frac{g^{\prime }}{g}$.

When the accelerometer or the inclinometer is used for measuring the gravity, as the output represents the weight of the earth gravitational field along the axis (Figure 3), the tilt angle of the axis with respect to the horizontal plane can be obtained easily via a sine function [11].

## 3. Acquiring Sensor Motion Using Inertial Navigation Algorithm

During the monitoring, a 3-axis accelerometer returns three values in each measurement, representing the respective weights of the earth’s gravitational acceleration along the three axial directions. In this sense each axis can be seen and handled equally to an independent sensor, like the two orthogonal 1-axis inclinometers which provide from each sensor a perpendicular inclination. Processing output, the tilt angle of each axis of the accelerometers and the inclinometers can be deduced. However, we cannot gain an understanding of the spatial variations of the sensors by only comparing the three values from accelerometers and the two values from inclinometers over time. Indeed, there is lack of a unified spatial parameter that enables a comparison of the results from distinct sensors. Hence, before the study of the spatial variations of the sensors, a normal vector should be calculated as a standard expression, representing the position of a sensor in a three dimensional Cartesian coordinate system. The deviation of the normal vector can be divided into three gradual steps [15] and will be briefly explained as follows.

### 3.1. Expression of g’

**Figure 4:**
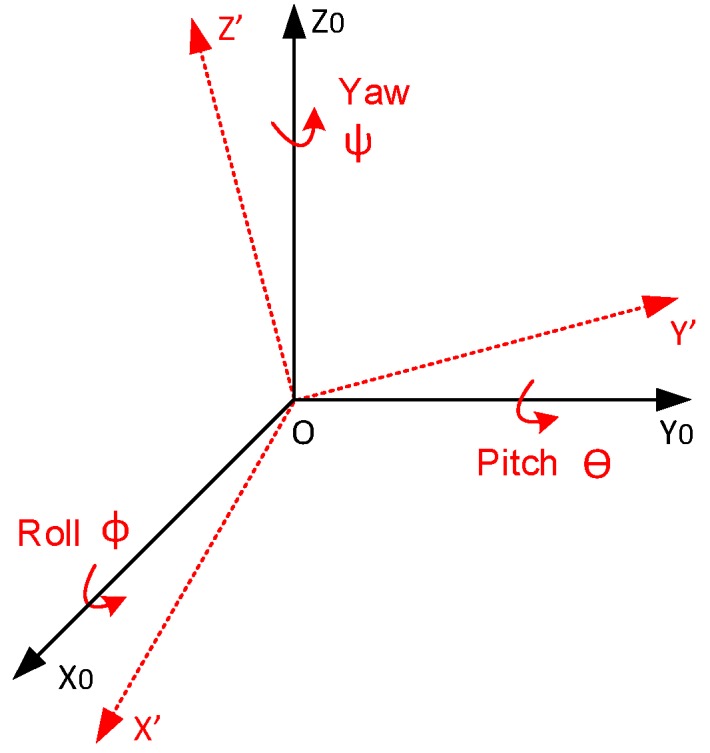
Coordinate System Transforming Through the Order of X-Y-Z (modified from [11]). The initial coordinate system $OX_{0}Y_{0}Z_{0}$ is transformed to $OX^{\prime }Y^{\prime }Z^{\prime }$ considering the roll, pitch and yaw angles around the x-, y- and z-axis respectively.

We define a coordinate system $OX_{0}Y_{0}Z_{0}$, which is transformed to a new coordinate system OX′Y′Z′ by rotating a roll angle *ϕ*, a pitch angle *θ* and a yaw angle *ψ* around x-, y- and z-axis respectively (Figure 4). For each step of rotation, there is a corresponding rotation matrix R that transfers the expression of a vector based on the old coordinate system to an expression based on the new one. There are six possible orders for the rotation of the coordinate system but only the orders of Z-Y-X or Z-X-Y can assure a solution, since a spatial vector has two degrees of freedom [14]. Taking the Z-Y-X order for instance, after being rotated successively around z-, y- and x-axis, the rotated vector of earth gravitational acceleration (0,0,−1)′ can be expressed as:

(1) $$g\prime_{xyz}=R_{x}\left(\varphi \right)R_{y}\left(\theta \right)R_{z}\left(\psi \right)\left(\begin{array}{c} 0\\ 0\\ -1 \end{array}\right)=\left(\begin{array}{c} sin\theta \\ -cos\theta sin\varphi \\ -cos\theta cos\varphi \end{array}\right)$$

### 3.2. Acquirement of Rotation Angles

In this step, based on the equivalent relations between the sensor output value and the corresponding components of the g′ vector, the roll φ and pitch θ can be resolved and rotation matrices are hereby obtained. As a result of the mechanism of an accelerometer, the vector of the earth’s gravitational acceleration is exported as ${\left(0,0,1\right)}^{T}$ instead of ${\left(0,0,-1\right)}^{T}$ [11]. Similarly, the components of $g\prime_{xyz}$ are correspondingly equal to the magnitude of the normalized accelerometer reading $G_{p}{\left(G_{px},G_{py},G_{pz}\right)}^{T}$ after multiplying with −1, which can be presented as:

(2) $$\frac{G_{p}}{∥G_{p}∥}=-{g^{\prime }}_{xyz}=-\left(\begin{array}{c} sin\theta \\ -cos\theta sin\varphi \\ -cos\theta cos\varphi \end{array}\right)$$

Solving the equation, the roll φ and the pitch *θ* can be expressed as:

Accelerometer:

(3) $$\varphi =arctan\left(\frac{G_{py}}{G_{pz}}\right)$$

(4) $$\theta =-arcsin\frac{G_{px}}{\sqrt{G_{px}^{2}+G_{py}^{2}+G_{pz}^{2}}}$$

Inclinometer:

(5) $$\varphi =arcsin\frac{G_{py}}{\sqrt{1-G_{px}^{2}}}$$

(6) $$\theta =-arcsinG_{px}$$

Since current monitoring system is not equipped with a magnetometer or a gyroscope, the value of yaw *ψ* cannot be solved. Nevertheless, taking account of the relatively simple deformation mode in geotechnical projects and the slight relevance to construction safety, the yaw *ψ* that represents the rotation around z-axis could be considered as zero.

### 3.3. Derivation of the Normal Vector

After retrieving the expression of rotation matrices, we operate the matrices inversely and intend to convert the normal vector from the transformed coordinate system into a description according to the old one. Defining the upward direction that is perpendicular to the sensor board plane as the normal direction; following the Z-Y-X order, the equation that describes the correlation between initial normal vector *V_n_* and the converted normal vector ${\left(0,0,1\right)}^{T}$ in the rotated coordinate system can be expressed as:

(7) $$R_{x}\left(\varphi \right)R_{y}\left(\theta \right)R_{z}\left(\psi \right)V_{n}=\left(\begin{array}{c} 0\\ 0\\ 1 \end{array}\right)$$

As the rotation matrices $R_{z}\left(\psi \right)$, $R_{y}\left(\theta \right)$ and $R_{x}\left(\varphi \right)$ are unit orthogonal matrices (Unit Orthogonal Matrix: is a unitized square matrix, the transpose of which equals to its inverse, which can be expressed as $Q^{T}=Q^{-1},QQ^{T}=Q^{T}Q=E$, where *E* is the identity matrix), they satisfy the principle that the transpose equals to their inverse. Hence *V_n_* can be solved by:

(8) $$V_{n}=R_{z}^{T}\left(\psi \right)R_{y}^{T}\left(\theta \right)R_{x}^{T}\left(\varphi \right)\left(\begin{array}{c} 0\\ 0\\ 1 \end{array}\right)$$

(9) $$\Rightarrow V_{n}=\left(\begin{array}{c} cos\varphi sin\theta \\ -sin\varphi \\ cos\varphi cos\theta \end{array}\right)$$

The normal vector represents the tilt direction and the tilt angle of the sensor board, which moves along with the monitored objects during geotechnical events.

## 4. Positional Estimate Using Kalman Filter

The normal vectors calculated from raw data of the MEMS sensor have inherited interferences and noises from different sources and thus should not be used directly to represent the position. Therefore, Kalman filter is used subsequently to estimate the state of the observations. In particular, this allows also to apply the concept of sensor fusion if more than one sensor is used e.g., to monitor inclination or acceleration.

Kalman filter goes back on 1960, when R. E. Kalman proposed a recursive solution to the discrete-data linear filtering problem [16]. As one of the primary developers of the linear quadratic estimation (LQE), this method is named after his name. Kalman filter is a set of mathematical equations that uses a recursive means to estimate the underlying system state by minimizing the mean square error [17]. Nowadays, Kalman filter and its extended version have been widely used in target tracking, navigation, and other relevant fields in data processing [18,19]. In the following a brief introduction of Kalman filter followed by his application to our problem of noisy positional data is given.

### 4.1. A Brief Introduction of Kalman Filter

The first step of the Kalman filter is to build a model that represents the series of data. The state variable *x* is addressed as an expression of discrete linear stochastic difference equation and the measurement value *z* is described as a linear function of *x*:

(10) $$x_{k}=Ax_{k-1}+Bu_{k-1}+w_{k-1}$$

(11) $$z_{k}=Hx_{k}+v_{k}$$

where:

A—State transition matrix relating the state at the previous time step *k* − 1 to that at the current step *k*;
*u*—Optional control input;
B—Control matrix that relates *u* to the state *x*;
*w*—Process noise vector, *w*(*i*) is normally distributed denoted by *N*(0, *Q*) and the covariance $cov\left[w\left(i\right),w\left(j\right)\right]=Q\cdot \delta_{ij}$, where $\delta_{ij}=\{\begin{array}{c} 0\;if\;i\neq j\\ 1\;if\;i=j \end{array}$;
*H*—Meaurement transition matrix relating the state *x* to the measurement *z*;
*v*—Measurement noise vector, v(i) is normally distributed denoted by N(0,R) and the covariance $cov\left[v\left(i\right),v\left(j\right)\right]=R\cdot \delta_{ij}$, where $\delta_{ij}=\{\begin{array}{c} 0\;if\;i\neq j\\ 1\;if\;i=j \end{array}$, and w and v are statistically independent.

**Figure 5:**
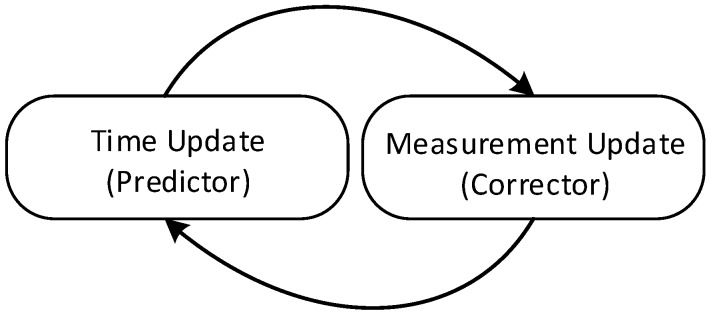
Kalman Filter Predictor-Corrector Cycle. The time update projects the current state estimate ahead, and then the measurement update adjusts the projected estimate based on the actual measurement.

Once the Kalman filter model is established, the estimating process is applied by a predictor-corrector cycle. As is shown in Figure 5, when the initial state value is established, the iteration can be immediately started. Specifically, time update equations and measurement update equations are respectively grouped that are responsible for the priori estimate for the next time step and the feedback of the priori estimate. Table 2 provides the update equations from groups of time and measurement. A detailed explanation of Kalman filter can be found in [17,20]. There are several formulations Kalman filter update equations; in this paper, a specific form of Kalman gain K and a posteriori estimate error covariance P are selected on purpose of a concise expression of Kalman filter algorithms (Table 2 and Table 3) for both single and multi-sensor systems. More information can be found in [21].

**Figure 6:**
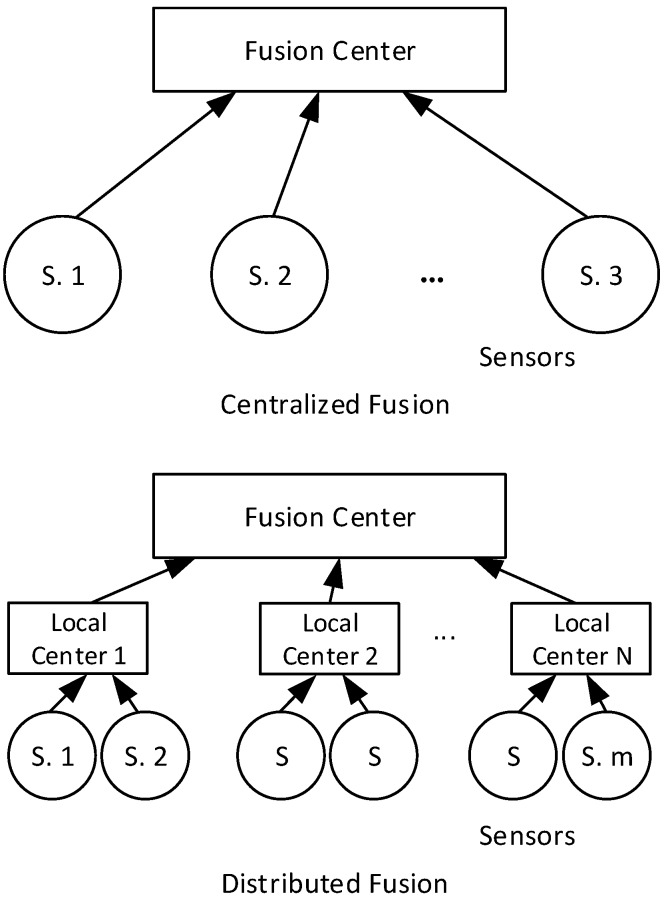
Centralized Fusion and Distributed Fusion. In centralized fusion, all measurements are sent to the fusion center; while in distributed fusion, the sensors supply data to a set of local processors to conduct pretreatment firstly, and then the result will be sent to the fusion center for a global estimate.

In a multi-sensor system, the main fusion methods include centralized and distributed means [22], the sketches of which are illustrated in Figure 6. Specifically, for centralized fusion methods, Willner, Chang [23] has introduced three main linear Kalman filter algorithms for a multi-sensor system, namely parallel filter, sequentially filter and data compression filter, and all the three filters are equivalent and optimum. For distributed fusion methods, Carson has proposed a federated Kalman filter as a typical representation [24,25]. Generally speaking, the centralized fusion consumes intensive resources during calculating but assures a limited loss of information, while the distributed fusion calculates faster but has a less accuracy. In the following application, the centralized fusion method will be applied considering the relatively small amount of processed data at the same time.

### 4.2. Application of Kalman Filter to Monitor Tunneling-Induced Ground Subsidence from Sensor Motion

**Figure 7:**
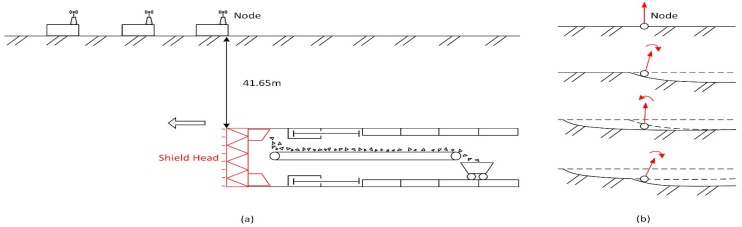
Schematic Diagram of Spatial Relation Between Sensors and the Shield Machine (**a**) and Expected Motion Stages of a Sensor Node Caused by Shield Tunneling (**b**) (modified from [11]).

A one-day field test of the X-SLEWS wireless sensor network was conducted from 10 a.m. to 5 p.m. on 27 January in 2013 at a tunneling construction site in Shanghai. The sensor nodes were deployed at the surface above the underground super highway tunnel at South Hongmei Road, while the shield machine would pass below the area at a depth of 41.65 m. Due to the subsidence related to the tunnel excavation, subsurface deformations was expected to monitor. Each node contains a 3-axis accelerometer and two orthogonal 1-axis inclinometers, and both of them are able to measure the tilting of the sensor with a settable frequency to the base station via a wireless network [8]. The MEMS sensors of the sensor node are orientated on a plane defined as sensor plane, and we observe the change of the orientation of the normal vector and define it as the expected motion due to the tunnel driving. Figure 7 shows the orientation of the MEMS sensor nodes in relation to the tunnel axis as well as the expected stages of motion caused by shield tunneling. When the excavation causes ground subsidence, the sensor node will firstly incline to the shield head, secondly restore to a certain extent with the expansion of the settlement area, and then tilt to the shield head again following the succeeding subsidence.

**Figure 8:**
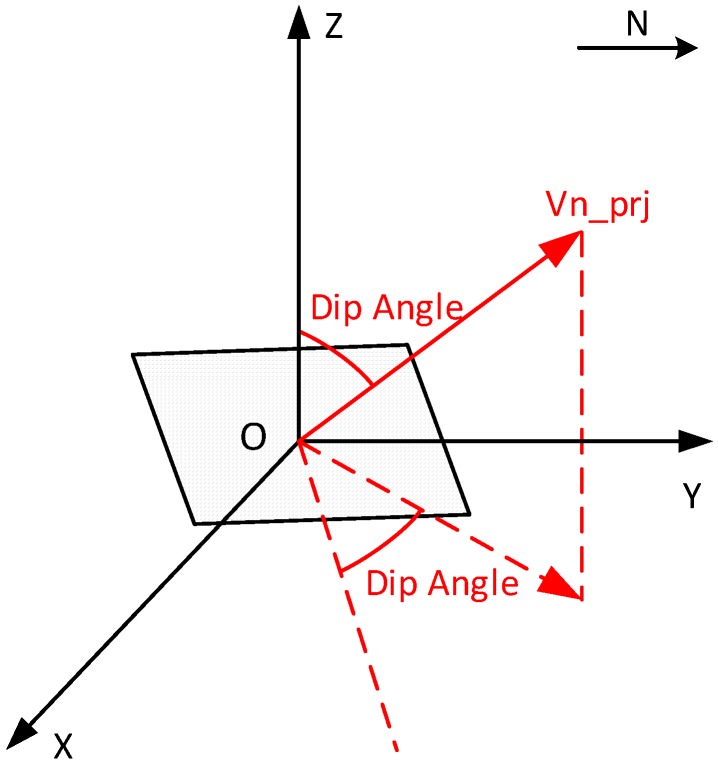
Derivation of the Dip Angle From the Projected Normal Vector $V_{n\_prj}$ in the Longitudinal Section (modified from [15]). The dip is the angle between $V_{n\_prj}$ and z positive semi-axis, as well as the angle between the sensor board and the horizontal plane.

**Figure 9:**
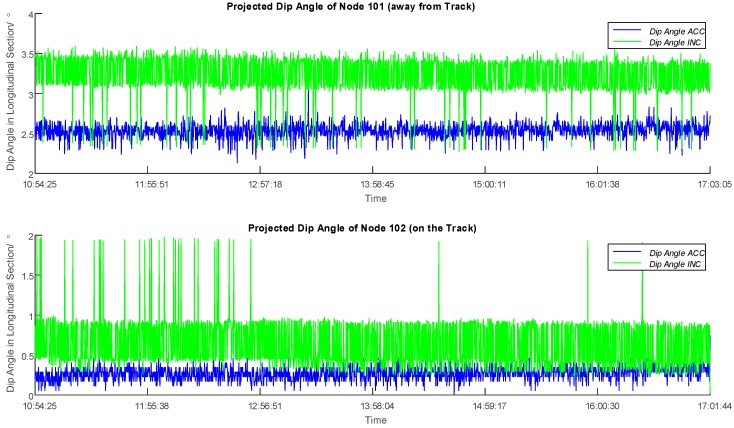
Plots of Dip Angles of Sensor Node 101 (away from Track) and Node 102 (on the Track) in the Longitudinal Section over the Observing Day. Blue and green lines stand for the performances of the accelerometer and the inclinometer. Vertical bars can be recognized as outliers.

The data retrieved from two sensor nodes have been processed in this paper as an example. Firstly, the normal vectors over time have been derived as briefly described above and in detail in [11]. To monitor the ground deformation, the change in inclination of the surface, respectively the sensor node, as depicted in Figure 7, can be used as a measure and to visualize the changes over time. The normal vectors have been consequently projected into the longitudinal section of the tunnel, and the dip angle values have been hereby solved to express the vertical the best tilting of the sensor. Figure 8 illustrates the derivation of the dip angle from the projected normal vector $V_{n\_prj}$ in the longitudinal section, and Figure 9 shows the plots of dip angles of two sensor nodes located away from and on the track in the longitudinal section over the observing day. Particularly, Node 102 was deployed on the surface on the track of the tunnel for the purpose of an observation of ground subsidence, while Node 101 located 16 m away from the excavation center is used as a reference of Node 102.

The change of the inclination has been simplified as a uniform motion. Hereby, the movement model of the inclination of the sensor node can be defined as:

(17) $$\theta_{k}=\theta_{k-1}+T\omega_{k-1}+w_{k-1},\;k=1,2,\ldots$$

(18) $$\omega_{k}=\omega_{k-1}+w_{k-1},\;k=1,2,\ldots$$

In which $\theta_{k}$ and $\omega_{k}$ are the tilt angle and the angular velocity at time k, T is the measuring time interval, $w_{k}$ is the process noise vector at time k.

During the monitoring, the measurements were conducted every ten seconds, thus the measuring time interval T is taken as 10. Therefore, the Kalman filter model is built as:

(19) $$x_{k}=Ax_{k-1}+\delta w_{k-1},\;k=1,2,\ldots$$

where $x_{k}=\left[\begin{array}{c} \theta_{k}\\ \omega_{k} \end{array}\right]$, $A=\left[\begin{array}{cc} 1 & 10\\ 0 & 1 \end{array}\right]$, $\delta =\left[\begin{array}{c} 1\\ 1 \end{array}\right]$, w is the process noise vector and it is normally distributed denoted by N(0,Q) and the covariance $cov\left[w\left(i\right),w\left(j\right)\right]=Q\cdot \delta_{ij}$, where $\delta_{ij}=\{\begin{array}{c} 0\;if\;i\neq j\\ 1\;if\;i=j \end{array}$.

Because the state transition matrix A has a high reliability, the result will not be influenced much by w, hence we define a relatively small value of covariance as 10^−7^ and thus $Q=\left[\begin{array}{cc} 0.0000001 & 0\\ 0 & 0.0000001 \end{array}\right]$.

Since there are two types of sensors (accelerometers and an inclinometers) have been embedded in a sensor node, both of the measurements are going to be processed in the fusion center. Hereby the measurement value z known as the tilt angle at step k is defined as:

(20) $$z_{k}=Hx_{k}+v_{k},\;k=0,1,2,\ldots$$

In which $z_{k}=\left[\begin{array}{c} z_{k,1}\\ z_{k,2} \end{array}\right]$, $H=\left[\begin{array}{c} H_{1}\\ H_{2} \end{array}\right]=\left[\begin{array}{cc} 1 & 0\\ 1 & 0 \end{array}\right]$, $v_{k}=\left[\begin{array}{c} v_{k,1}\\ v_{k,2} \end{array}\right]$. Assuming the measurement noise vector v is white noise with a covariance of 1, and thus $R_{k}=\left[\begin{array}{c} R_{k,1}\\ R_{k,2} \end{array}\right]=\left[\begin{array}{c} 1\\ 1 \end{array}\right]$.

The initial values of the model are defined as $x_{0}=\left[\begin{array}{c} 0\\ 0 \end{array}\right]$, $P_{0}=\left[\begin{array}{cc} 1 & 0\\ 0 & 1 \end{array}\right]$. These values are substituted into Equation (21) to Equation (25) in Table 3, following the Kalman filter cycle as shown in Figure 5.

**Figure 10:**
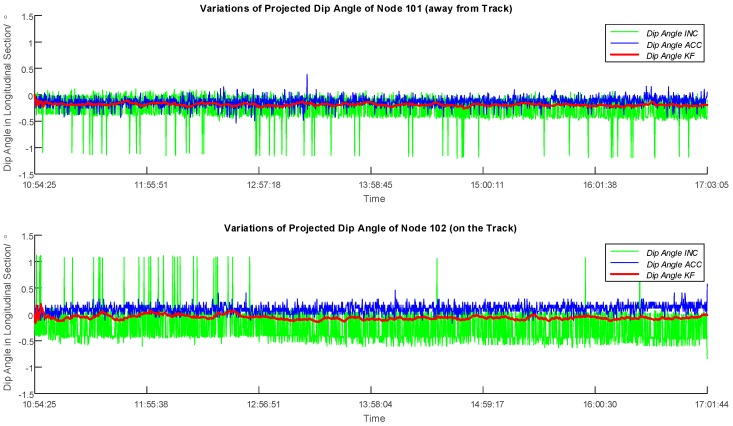
Plots of Dip Angle Variations of Sensor Node 101 (away from Track) and Node 102 (on the Track) in the Longitudinal Section over the Observing Day. The absolute values were subtracted by the initial value to obtain variations of dip angles, and the changes detected from the accelerometer and inclinometer can thus be compared directly. Blue and green lines stand for the performances of the accelerometer and the inclinometer, and the red line is the result after the application of Kalman filter. Vertical bars can be recognized as outliers.

Before the application of Kalman filter, the values of dip angles of Nodes 101 and 102 were subtracted by their initial values individually, as there was an initial offset for each sensor due to the soldering. In this way the variation of dip angles of both sensors of Nodes 101 and 102 can be plotted, which are presented in Figure 10 together with the filtered result. In general, the filtered dip angles show much lower variances compared to the values from the accelerometer and the inclinometer. A discussion and evaluation is given in the following.

### 4.3. Evaluation and Discussion of the Filtering

As is shown in Figure 10, vertical bars in blue and green indicate outliers caused by noise interferences from electrical fields and ground vibration when the tunnel boring machine and other construction measures approach, where the sensor node would be affected and thus would obtain inaccurate measurements. Regarding the accelerometer, the combination of the three 1-axis accelerometer has enabled a normalization in calculating the normal vector, but the relatively low sensitivity of 0.0637°/count has reduced the reliability of the deduced dip angle. As for the inclinometer, in spite of the high sensitivity of 0.00179°/count compared to the accelerometer, the output from the inclinometer could not be normalized because only two orthogonal axes were deployed with inclinometers. Therefore, to carry out a data filter to identify the outliers on the observed data before they are used for an assessment is necessary and valuable. In Figure 10, the scale of the outliers for the inclinometer is evidently much bigger than that of the accelerometer for both nodes. Hence, it can be inferred that with a strong effect of noise interferences and ground vibration, the difference of sensitivity would not be the main factor of malfunction any more. Instead, the defect of normalization of the inclinometer has resulted in higher ratio of outliers and an unstable performance.

**Figure 11:**
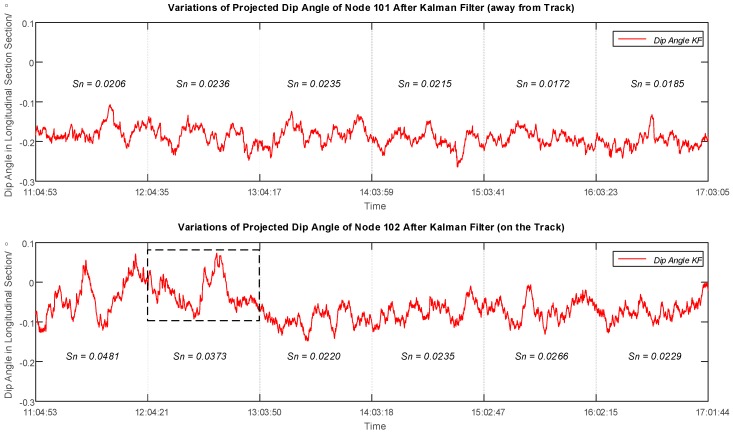
Plots of Dip Angle Variations after Kalman Filter of Sensor Node 101 (away from Track) and Node 102 (on the Track) in the Longitudinal Section over the Observing Day with Hourly Standard Deviations. The values of standard deviations are mostly between 0.0172 and 0.0266, and the two exceptions appear in the first two hours for Node 102 indicating a disturbance of the data.

To explore more information hidden from the variations of dip angles, standard deviations of the filtered dip angles were calculated every hour for both sensor nodes, and amplified filtered variations of dip angles with sectional standard deviation values is presented in Figure 11. The standard deviation values are mostly between 0.0172 and 0.0266, but two exceptions appear in the first two hours for Node 102 with a value of 0.0481 and 0.0373 respectively. On the other hand, Node 101 that was deployed 16 meters away performed regularly in the first two hours compared to its latter performance. Hereby, the abnormality of Node 102 indicates a disturbance of the data when the tunnel boring machine passed through the area underground.

**Figure 12:**
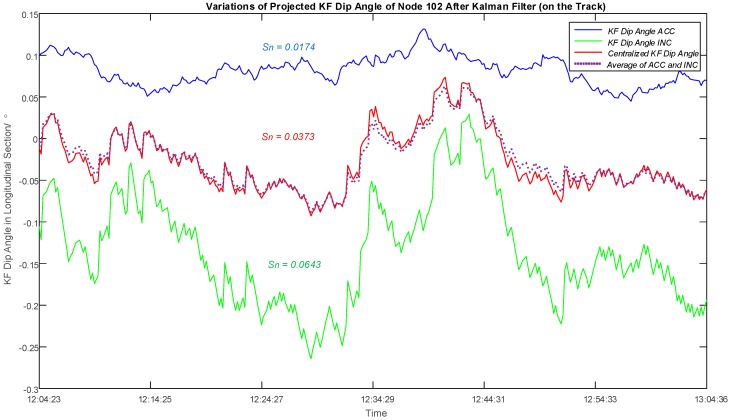
Plots of Dip Angle Variations after Kalman Filter of Node 102 (on the Track) in the Longitudinal Section During the Second Measuring Hour. Blue and green lines are results after Kalman filter for the accelerometer and inclinometer respectively, and the red line shows the result solved by a centralized Kalman filter based on the tilt values from two sensors. The standard deviation of the red line is higher than that of the blue line and lower than that of the green line. The purple dot line displays the average of the blue and green lines.

Taking the second measuring hour for Node 102 as an example, a comparison was made between the filtered dip angles based on different algorithms of Kalman filter. Figure 12 shows the plots of dip angle variations over time after distinct filtering. The blue line corresponds to the filtered dip angles based on measurements from the 3-axis accelerometer, while the green line represents the result corresponding to the two orthogonal 1-axis inclinometers. The red line shows centralized filtered dip angles on the basis of both the accelerometer and the inclinometer, and the average of two individual filtering (blue and green line) is also indicated with purple dot line. It can be observed that the centralized filtering is not always consistent with the curve of the average. The standard deviation of the inclinometer line is much higher than the accelerometer line, which indicates that the lack of z-axis of the inclinometer combination has led an instable performance. Nevertheless, considering its higher sensitivity, the variations calculated by inclinometers should still be taken into account, especially when the variations of dip angle are not notable. The standard deviation of the dip angle variations based on both sensor values is higher than that from the accelerometer and lower than that from the inclinometer, which have combined the advantages from both sensors and reduced to the instability to a certain level.

**Figure 13:**
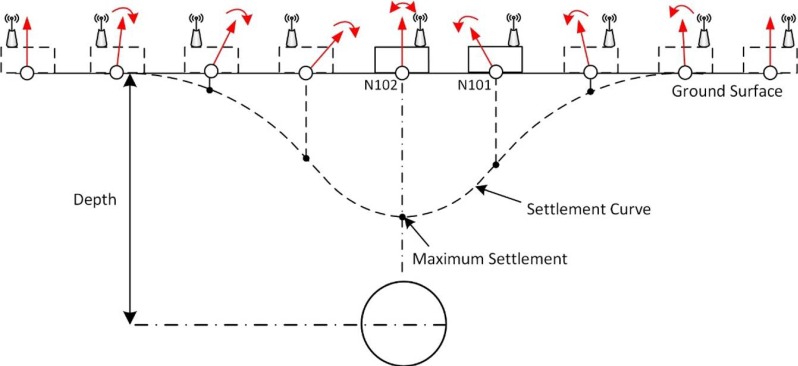
Planned Distribution of Sensor Nodes along the Transversal Direction and Drawing of a Settlement Curve. The settlement is gradually accumulated from the node deployed at a distance showing no variations to the one at the excavating center on the ground. Starting with a value of zero, a smooth curve of vertical ground settlement can be obtained using the tangent function and interpolation.

If the sensor node was deployed exactly on the ground above the tunneling center, it is not possible to evaluate the ground settlement by just calculating the tilt angle from only one single node located at the center along the longitudinal or the transversal direction. Nevertheless, when a series of sensor nodes are closely located on the ground from a distance away to the excavating center, a smooth subsidence map can be achieved. Figure 13 presents a sketch showing the way of deploying sensor nodes in order to draw a settlement curve in the cross section. Simplifying the subsided ground surfaces between two adjacent sensor nodes are even, the absolute ground subsidence in the section can be calculated via the tangent function presented as a polyline. After that, a smooth subsidence curve can be obtained by interpolating the polyline (Figure 13). Anyhow, it has to be considered that modern tunnel driving in urban areas and especially in the Shanghai areas is carried out with large experience and considering larger safety margins to reduce settlements. Therefore, ground displacements and tilt motion are always considerably low.

Finally, as Kalman filter is a recursive solution that can be continually applied to estimate the situation in the next time step and then be updated after obtaining the observation, the a priori estimate made by Kalman filter can be taken into account into a Building Information Model (BIM) to support the management and facility operation. Furthermore, it can be used as a kind of early warning systems to indicate changes in the ground response to the excavation.

## 5. Conclusions

In this paper, a one-day field test was deployed on top of a tunnel section under construction and MEMS sensor nodes equipped with both accelerometers and inclinometers were used to measure the changes of the surface caused by excavation. Normal vectors of the sensors were deduced using rotation matrices and consequently were projected to the longitudinal section for the purpose of an assessment of ground settlement. Aiming at an improved data reliability and a better estimate of the inclination, a centralized Kalman filter was conducted based on the results from accelerometers and inclinometers. Furthermore, a discussion based on the filtered result was provided afterwards to evaluate the performance of the sensors and the process of tunneling.

## Figures and Tables

**Table 1:** Generic Data Sheet of the two Sensors Used in the Deployment.

Sensor Type	Accelerometer	Inclinometer
Product	SCA3100-D04	SCA830-D07
Size(w × h × l)	7.0 × 3.3 × 8.6 mm^3^	7.6 × 3.3 × 8.6 mm^3^
Measurement Axis	3-axis	1-axis
Range	±2 g	±1 g
Sensitivity (LSB/g)	900 (0.0637°/count)	32,000 (0.00179°/count)

**Table 2:** Kalman Filter Update Equations (${\hat{x}}_{k}^{-}$—A priori state estimate at step k, $P_{k}^{-}$ —A priori estimate error covariance at step k, ${\hat{x}}_{k}$—A posteriori state estimate at step k, $P_{k}$—A posteriori estimate error covariance at step k, $K_{k}$—Kalman Gain that is deduced by minimizing $P_{k}$).

Time Update Equations		Measurement Update Equations	
${\hat{x}}_{k}^{-}=A{\hat{x}}_{k-1}+Bu_{k-1}$	(12)	$K_{k}=P_{k}H^{T}R_{k}^{-1}$	(14)
$P_{k}^{-}=AP_{k-1}A^{T}+Q_{k-1}$	(13)	${\hat{x}}_{k}={\hat{x}}_{k}^{-}+K_{k}\left(z_{k}-H{\hat{x}}_{k}^{-}\right)$	(15)
		$P_{k}={\left(P_{k}^{-}\;{}^{-1}+H^{T}R_{k}^{-1}H\right)}^{-1}$	(16)

**Table 3:** Kalman Filter Equations for Centralized Fusion.

Time Update Equations		Measurement Update Equations	
${\hat{x}}_{k}^{-}=A{\hat{x}}_{k-1}$	(21)	$K_{k,i}=P_{k}H_{i}^{T}R_{k,i}^{-1}$	(23)
$P_{k}^{-}=AP_{k-1}A^{T}+Q_{k-1}$	(22)	${\hat{x}}_{k}={\hat{x}}_{k}^{-}+\overset{2}{\underset{i=1}{\sum }}K_{k,i}\left[z_{k,i}-H_{i}{\hat{x}}_{k}^{-}\right]$	(24)
		$P_{k}={\left(P_{k}^{-}\;{}^{-1}+\overset{2}{\underset{i=1}{\sum }}H_{i}^{T}R_{k,i}^{-1}H_{i}\right)}^{-1}$	(25)
